# Influence of Inter-Set Stretching on Strength, Flexibility and Hormonal Adaptations

**DOI:** 10.2478/hukin-2013-0013

**Published:** 2013-03-28

**Authors:** Antônio Claudio Souza, Claudio Melibeu Bentes, Belmiro Freitas de Salles, Victor Machado Reis, José Vilaça Alves, Humberto Miranda, Jefferson da Silva Novaes

**Affiliations:** 1 Research Center for Sport, Health, and Human Development (CIDESD). University of Trás-os-Montes and Alto Douro (UTAD), Vila Real, Portugal.; 2 Universidade Federal do Rio de Janeiro. School of Physical Education and Sports. Rio de Janeiro – Brazil.

**Keywords:** strength performance, static stretching, cortisol, growth hormone, flexibility

## Abstract

Adequate levels of strength and flexibility are important for the promotion and maintenance of health and functional autonomy as well as safe and effective sports participation. The aim of the present study was to analyze the effects of 8 weeks of strength training with or without inter-set static stretching on strength, flexibility and hormonal adaptations of trained men. Sixteen trained men were randomly divided into 2 groups: the static stretching group (SSG) and passive interval group (PIG). All participants performed 24 training sessions 3 times a week. The test and retest of 8RM, strength, flexibility, cortisol and growth hormone concentration in pre and post test conditions were also evaluated. To compare the differences between and within groups in pre- and post-training tests, ANOVA with repeated measures was performed (SSG_pre_ x SSG_post_; PIG_pre_ x PIG_post_; SSG_post_ x PIG_post_). An alpha level of p<0.05 was considered statistically significant for all comparisons. Both groups showed significant increases in strength (SSG_pre_ vs. SSG_post_; PIG_pre_ vs. PIG_post_) in the same exercises for leg extension (LE) and Low Row (LR). Specifically, in the SSG group, the parameters for LE were (p = 0.0015 and ES = 2.28 - Large), and the parameters for LR were (p = 0.002 and ES = 1.95 - Large). Moreover, in the PIG group, the parameters for LE were (p = 0.009 and ES = 1.95 - Large), and the parameters for LR were (p = 0.0001 and ES = 2.88 - Large). No differences were found between the groups (SSG_post_ vs. PIG_post_). Both groups showed significant increases in flexibility but in different joints (SSG_pre_ vs. SSG_post_; PIG_pre_ vs. PIG_post_). In the SSG group, only three joints showed significant increases in flexibility: shoulder extension (p = 0.004 and ES = 1.76 - Large), torso flexion (p = 0.002 and ES = 2.36 - Large), and hip flexion (p = 0.001 and ES = 1.79 -Large). In the PIG group, only three joints showed increases in flexibility: horizontal shoulder abduction (p = 0.003 and ES = 2.07 - Large), hip flexion (p = 0.001 and ES = 2.39 – Large), and hip extension (p = 0.02 and ES = 1.79 - Large). In-between group analyses (SSG_post_ x PIG_post_) revealed differences in two joints: shoulder extension (p = 0.001) and horizontal shoulder abduction (p = 0.001). Hormonal profiles showed no significant differences in cortisol secretion or growth hormone concentration. In conclusion, both studied strength protocols (with and without inter-set static stretching) resulted in flexibility and strength gains without an effect on the anabolic and catabolic hormonal profile.

## Introduction

Adequate levels of strength and flexibility are important for the promotion and maintenance of health and functional autonomy, as well as safe and effective sports participation (ACSM, 1998; Simão et al., 2011). In this context, strength training (ST) is considered an integral component of a well-rounded exercise program, contributes to the treatment and prevention of injuries, and improves sports performance (ACSM, 2002; ACSM, 2009).

The combinations of different types of stretching modes on athletic performance have been previously studied (Mikolajec et al., 2012; Shrier, 2004; Bacurau et al., 2009; Beckett et al., 2009; Little and Williams, 2006; Yamaguchi and Ishii, 2005; Behm et al., 2001; Dalrymple et al., 2010). All of these studies, with the exception of the study by Dalrymple et al. (2010), observed a decrease in explosive sport skills, such as sprinting and vertical jumps. However, Dalrymple et al. (2010) did not explain the influence of the two different stretching models (passive and dynamic stretching) on the countermovement jump.

Gomes et al. (2010) observed a decrease in the capacity to maintain force on strength training exercises before proprioceptive neuromuscular facilitation (PNF). In this study, static stretching did not affect endurance or strength performance.

Research has also demonstrated that a different inter-set rest interval length can produce different acute responses and chronic adaptations in neuromuscular and endocrine systems (Salles et al., 2009). However, little research has focused on the activity performed during these recovery periods (Caruso and Coday, 2008; Garcia-Lopez et al., 2010). It is common to see lifters performing ST inter-set stretching to improve the muscular recovery in sports or recreational-related exercises (Garcia-Lopez et al., 2010). Additionally, it has been suggested that inter-set stretching influences the time under tension and associated neuromuscular, metabolic, and/or hormonal systems. Recent data have shown that ST inter-set static stretching negatively affected the bench press acute kinematic profile compared with inter-set ballistic stretching and non-stretching conditions (Garcia-Lopez et al., 2010). In a chronic manner, static stretching performed before ST sessions resulted in similar strength gains to ST alone, suggesting that strength and stretching can be prescribed together to achieve optimal improvements in flexibility (Simão et al., 2011). Based on these results, the performance of inter-set static stretching may lead to additional improvements in flexibility levels and muscular recovery without additional time expended in the gym.

However, to date, only Simão et al. (2011) have observed the chronic effects of ST inter-set stretching on flexibility. Therefore, the aim of the present study was to analyze the effects of eight weeks of strength training with and without inter-set static stretching on strength, flexibility and hormonal adaptations.

## Material and Methods

### Participants

The initial sample was composed of 16 trained men. All participants underwent a routine clinical evaluation. To be included in the experiment, volunteers had to meet the following criteria: (a) be trained for at least 24 months with a weekly frequency of three days; (b) agree to not perform any type of regular physical activity other than the prescribed strength training and flexibility training during the experiment; (c) be free from any condition that would influence the collection or interpretation of data; and (d) be free from the intake of ergogenic aids that could influence the collection or interpretation of the data. The 16 men were randomly assigned to 2 groups: the static stretching group (SSG; n = 8) or passive interval group (PIG; n = 8) (Table 1). Study details were explained verbally and in writing, and all participants signed an informed consent form before participation in the study in accordance with the declaration of Helsinki. The study protocol was approved by the Research Ethics Committee of the State University of Pará (Brazil).

### Eight Repetition Maximum Test (8RM)

After a strength training familiarization period (4 sessions), all participants performed 2 familiarization sessions with the 8RM test protocol, with 72 hours between sessions. The 8RM tests were performed for the following exercises: machine bench press (BP), leg extension (LE), low row (LR), leg curl (LC), shoulder press (SP), and leg press (LP) (Techno Gym^®^-Gambettola, Italy) using a counterbalanced order. On day 1, the first 8RM test was performed, and then, after 72 hours, the 8RM test was repeated to determine test-retest reliability. The heaviest load achieved on the two test-retest days was considered the 8RM load. No exercise was allowed in the 72 hours between the 8RM tests. To minimize error during the 8RM tests, the following strategies were adopted (Simão et al., 2005; ACSM, 2010): (a) standardized instructions concerning the testing procedures were given to participants before the test; (b) participants received standardized instructions on exercise technique; (c) standard verbal encouragement was provided during the testing procedure; d) verbal stimuli were used to maintain a high exercise intensity; e) the additional weights used in the study were previously calibrated on a precision scale (Filizola, Brazil); f) for a repetition to be validated, a complete range of motion had to be performed. The 8RM was determined in fewer than 5 attempts, with a rest interval of 5 minutes between them; g) no pause was allowed between the eccentric and concentric phase of a repetition or between repetitions, and the velocity was controlled with a metronome (Qwik Time Quartz Metronome, Evets Corp., Laguna Beach, CA) calibrated to 60 beats × min^−1^.

After the 8 weeks of training, the 8RM test was performed with the same procedures of the pre-training test to observe the possible strength gains within and between groups. The 8RM tests were consistently conducted during the morning for each participant.

### Flexibility Measurement (Goniometry Protocol)

Flexibility was measured before and after 8 weeks of the experiment in 8 maximum stretching articular movements (ACSM, 2010). The flexibility measurements were taken 72 hours after the last 8RM test. The maximum flexibility measurement registered in 3 attempts with an interval of 10 seconds between attempts was considered for further evaluation (ACSM, 2011). The same procedure was executed post-training. All flexibility tests were conducted at the same time of the day. The data collected during the first evaluation were not made available to the evaluator to prevent information bias during measurements taken after training. Before the flexibility test, a warm-up was performed for the muscle groups involved in the evaluation. Two sets were used for the static stretching warm-up protocol, holding the position for 10 seconds in each set, until a point of slight discomfort was reached. A 10-second interval was provided between the warm-up stretching sets. The 8 maximum stretching articular movements were: a) shoulder flexion; b) shoulder extension; c) horizontal shoulder abduction; d) horizontal shoulder adduction; e) torso flexion; f) torso extension; g) hip flexion; and h) hip extension.

### Blood Evaluations (Cortisol and Growth Hormone)

Both the SSG (n = 8) and PIG (n = 8) group participants underwent two blood collections: one at baseline and the second at the end of the eighth week of the exercise program. Blood was collected by a trained professional (approximately 5-ml blood samples from the antecubital vein) at 8 am to avoid the different concentrations of the hormonal circadian rhythm, with 12 h of rest. Blood samples were shipped in conditions suitable for laboratory analysis. Growth hormone was analyzed using the chemiluminescent enzyme immunometric method, while cortisol was analyzed using a chemiluminescent enzyme immunoassay.

### Procedures

Before the 8-week training program (24 total sessions), 16 trained men were randomly assigned to 2 groups: the static stretching group (SSG; n = 8) and passive interval group (PIG; n = 8). The SSG and PIG groups performed 4 familiarization sessions with the exercises included in the training program. After familiarization with the exercises and before 8RM tests and retests, the subjects performed 2 sessions covering the 8RM procedures. Individuals performed the test and retest of 8RM, test and retest of flexibility and had their cortisol and growth hormone (GH) evaluated under the pre-test and post-test conditions. The baseline measurements of the hormonal responses, strength and flexibility tests were taken 72 hours apart. After the flexibility measurement, both groups underwent 8 weeks of training under the supervision of experienced fitness professionals. After the 8-week training program, flexibility, strength, and hormone concentrations were evaluated again.

### Training Protocols

The training protocol for all groups included 3 weekly sessions on alternate days, for a total of 24 sessions. All 16 subjects completed the study. All sessions were supervised by experienced fitness professionals. Strength training was composed of 6 exercises executed in 4 sets with 8RM. The order established for the strength training was as follows: BP, LE, LR, LC, SP, and LP. Before each training session, the subjects executed a specific warm-up involving 15 repetitions with 50% of the load used in the first and second exercises of the sequence. The rest interval between sets was 2 min and included passive rest or static stretching exercises for the muscle involved in the ST exercises for the PIG and SSG group, respectively. Static stretching was performed at the point of mild discomfort, with stretches held for 30 seconds (ACSM, 2011). The rest interval between an exercise was 5 min.

The initial training loads were adjusted for all participants and increased when the volunteers were able to perform more than 8RM by readjustment of 5% of the initial loads. All testing and training sessions were performed in the morning hours and were consistent throughout the study. To protect against bias, the investigators that conducted the training program did not conduct the testing measurements, and the staff involved in the strength and flexibility tests were blinded to the group assignment.

### Statistical Analyses

The statistical analysis was initially performed using the Shapiro–Wilk normality test and homoscedasticity test (Bartlett criterion). As mentioned, the 8RM tests were found to be similar when tested on two occasions prior to performing the different sequences. To compare the differences between and within groups in pre-training and post-training tests, ANOVA with repeated measures was performed (SSG_pre_ × SSG_post_; PIG_pre_ × PIG_post_; SSG_post_ × PIG_post_). An alpha level of p<0.05 was considered statistically significant for all comparisons. All statistical analyses were conducted using SPSS statistical software package, version 20.0 (SPSS Inc., Chicago, IL). The calculation of effect size (the difference between pretest and posttest scores divided by the pretest standard deviation) and scale proposed by Rhea (2004) were used to examine the magnitude of any treatment effect.

## Results

### Strength Results

The results obtained show an intraclass coefficient of SSG group: BP = 0.97; LE = 0.97; LR = 0.93; LC = 0.98; SP = 0.99; LP = 0.98 and in PIG group: BP = 0.96; LE = 0.98; LR = 0.80; LC = 0.94; SP = 0.97; LP = 0.98. A paired-samples t-test was performed and did not demonstrate any significant difference (p < 0.05) between 8RM tests on separate testing occasions. Both groups showed significant increases in strength (SSG_pre_ vs. SSG_post_ ; PIG_pre_ vs. PIG_post_), in same exercises; LE and LR. In SSG group in LE (*p* =0.0015 and ES = 2.28 - Large) and LR (*p =* 0.002 and ES = 1.95 -Large) and in PIG group in LE (*p =* 0.0090 and ES = 1.95 – Large) and in LR (*p =* 0.0001 and ES = 2.88 - Large).

No differences were showed between groups (SSG_post_ vs. PIG_post_). All results are presented in Table 2.

### Flexibility Measurements

Both Groups showed significant increases in flexibility, but in different joints (SSG_pre_ vs. SSG_post_; PIG_pre_ vs. PIG_post_). In SSG Group, only three joints showed significant increases in flexibility: shoulder extension (*p* = 0.004 and ES = 1.76 - Large); torso Flexion (*p* = 0.002 and ES = 2.36 – Large) and hip flexion (*p* = 0.001 and ES = 1.79 – Large). In PIG group, only three joints showed increases in flexibility: horizontal shoulder abduction (*p* = 0.003 and ES = 2.07 – Large); hip flexion (*p* = 0.001 and ES = 2.39 – Large) and hip extension (*p* = 0.02 and ES = 1.79 – Large).

In between groups analyses (SSG_post_ x PIG_post_) differences were found only in two joints: shoulder extension (*p* = 0.001) and horizontal shoulder abduction (*p* = 0.001). All results are presented in Table 3.

### Hormone profile

The results showed no significant differences in the concentration of cortisol and growth hormone (Table 4 – p > 0.05). Effect size data demonstrated trivial results in both hormones in SSG and PIG group (pretest vs. posttest).

## Discussion

The purpose of the present study was to analyze the effects of eight weeks of ST in 2 experimental groups (SSG and PIG) with and without inter-set static stretching on strength, flexibility and hormone profile of trained men. It was hypothesized that ST performed with inter-set static stretching would not result in additional strength and flexibility and also not change the anabolic-catabolic hormone profile after 24 weeks of training. The key finding of the present study was that both training groups presented significant strength and flexibility gains after 24 weeks and showed no differences in the anaboliccatabolic hormone profile, which confirmed the initial hypothesis. Additionally, the inter-set static stretching ST group demonstrated larger strength gains in two exercises and larger flexibility gains in three joints compared with ST alone. However, the results revealed a significant increase in muscle strength for only a few exercises in the SSG (LP, LR) and PIG (LR) experimental conditions.

This indicates that stretching between sets does not compromise increases in strength achieved by resistance training. Inter-set static stretching significantly changed the strength of LE in the SSG group and LR in both groups. These findings are consistent with previous studies on this issue (Nelson et al., 2005; Bacurau et al., 2009; Gomes et al., 2011) and indicate that stretching does not modify the strength gains promoted by resistance training. These findings have potentially important implications for strength and conditioning professionals who may commonly use stretching exercises as an integral part of a warm-up routine or during the training session itself. Perhaps, these results could be applied to the resistance training protocol with a long-term rest interval, 2 minutes between sets and 5 minutes between exercises and high loads with 8RM.

To our knowledge, this is the first study that analyzed the chronic effects of inter-set stretching. However, previous studies have analyzed the chronic effects of pre- (Simão et al., 2011) or post-ST (Nóbrega et al., 2005) stretching on strength and flexibility gains. Nevertheless, the effect size in the current study showed that strength training with stretching inter-set intervals increases flexibility in previously recreationally trained men, and strength and flexibility can be prescribed together to achieve better flexibility results. Indeed, Simão et al. (2011) analyzed strength and flexibility gains achieved through isolated or simultaneous strength and flexibility training in adult sedentary women. The sedentary women were randomly assigned to ST, flexibility training, the combination of both, or a control group. All of the groups performed preand post-training sit and reach tests to verify the flexibility level and 10RM tests for leg press and bench press exercises. The training protocol for all of the groups, except for the control group, was composed of three sets of eight exercises for upper and lower limbs three times per week. The flexibility training was composed of static stretching exercises that involved the upper and lower limbs performed before ST sessions. The results showed that the ST, ST + flexibility, and flexibility groups had significantly increased flexibility compared with baseline and the control group. The strength tests demonstrated that ST and ST + flexibility significantly increased 10RM when compared with baseline flexibility and the control group. The authors suggested that strength and flexibility can be prescribed in the same session to increase flexibility to a greater extent. Similar to our findings, the strength and flexibility training group presented larger flexibility gains than the ST alone group; however, there were no additional strength gains when compared with the ST alone group. Kokkonen et al. (2007) conducted research to verify the differences in lower limb strength gains in physically active individuals by comparing strength training in isolation versus strength training combined with static stretching exercises for the hip, thigh muscles and plantar flexors. They found significant strength increases in the lower limbs for both groups. However, the greatest differences were observed in the group that performed strength training in combination with stretching exercises (16%, 27% and 31% in the 1RM test for knee flexion, knee extension, and leg press exercises, respectively). The data from our study showed an increase of 27.79% in the knee extension exercise in the SSG group, which was in agreement with the study by Kokkonen et al. (2007). Moreover, the present study showed that increases in strength could be observed in the SSG group in the LR with an increase of 23.19% in the 8RM test; the PIG group demonstrated increases in strength in the LR (18.72%) in the 8RM test.

Mohamad et al. (2011) reported that stretching between sets of ST could increase muscle hypertrophy and suggested the possibility of additional increases in muscle strength in relation to strength training alone. However, it is important to emphasize that our study did not assess muscle hypertrophy, but only one anabolic growth hormone and one catabolic hormone (cortisol). These hormones showed no significant differences between pre- and post-ST evaluations between the groups. It is also important to note that the inclusion of stretching during intervals between sets did not reduce the gains at the end of the experiment or the intensity at which our participants were able to train. There are multiple studies that have suggested acute strength impairments following the performance of static stretching. This phenomenon could easily have affected chronic adaptations and daily session loading.

Any decrease in the acute training load could have contributed to the differing amounts of strength gains between the groups. Nóbrega et al. (2005) investigated the interaction of ST and flexibility training in young sedentary men and women. The subjects followed an ST protocol with intensities initially set at 60% of 1RM which was continuously adjusted so that fatigue was achieved after 8–12 repetitions. Static stretching exercises were performed after the ST sessions. At the end of 12 weeks, the authors verified that resistance training improved muscle strength either alone or in combination with flexibility training; however, ST alone did not change flexibility. Flexibility increased with specific training alone or in combination with ST. Similar to Simão et al. (2011) and our results, Nóbrega et al. (2005) found strength gains with ST alone and in combined strength and flexibility training groups.

In our study, the finding that larger strength increases in two exercises were observed in the inter-set stretching group is new. Previous literature has not presented larger strength gains with the inclusion of stretching exercises in a ST routine, which may be related to the fact that the static stretching exercises were performed between sets. Inter-set stretching will influence the time under tension and the associated neuromuscular, metabolic, and/or hormonal responses (Mohamad et al., 2011), which are related to the larger strength improvements presented by the inter-set stretching group.

The increased time under tension increases the effect of various neuromechanical and metabolic stimuli that are thought to be important for hypertrophic adaptation (Mohamad et al., 2011). It has been suggested that during ischemic conditions, such as during inter-set stretching, metabolites and ions accumulate rather than dissipate, which in turn leads to GH secretion and increased levels of IGF-1 (Mohamad et al., 2011). The limitations of our study include no inclusion of a control group, the short experimental period, and reduced adaptation to ST in the trained sample.

## Conclusions and Practical Implications

In conclusion, both studied ST protocols (with and without inter-set static stretching) resulted in flexibility and strength gains without influencing the anabolic-catabolic hormone profile. However, the results suggest that inter-set static stretching can be adopted to achieve additional strength and flexibility gains. Future studies analyzing the flexibility and strength gains in response to different inter-set stretching strategies, longer intervention periods, and different samples are necessary to confirm the results. Further research is also necessary to verify the effects of these strategies on hypertrophic adaptation, as previously suggested.

The results of the present study indicate that inter-set static stretching leads to additional improvements in strength and flexibility without additional time expended in the gym. The time saved by omitting separate stretching routines may help increase adherence to training by recreational fitness practitioners who have limited exercise time.

## Figures and Tables

**Table 1:** Characterization of the sample in pretraining situation. *ł

	**SSG (n = 8)**	**P-value (SW)**	**PIG (n = 8)**	**P-value (SW)**
**Age (y)**	**22.13 ±2.74**	**0.202**	**23.13 ± 1.55**	**0.731**
**Height (cm)**	**176.13± 1.65**	**0.423**	**176.88 ±2.30**	**0.168**
**Body mass (kg)**	**81.00 ± 7.95**	**0.055**	**85.13 ± 7.90**	**0.356**
**% Body fat**	**12.88 ± 1.79**	**0.447**	**13.90 ± 1.90**	**0.823**
**BMI (Kg·m^−2^)**	**26.37 ±2.00**	**0.341**	**27.18 ±2.02**	**0.206**

Legend: PSS = Passive stretching group; PIG = Passive interval group; BMI= Body mass índex; SW = Shapiro-Wilk.

**Table 2:** Strength results at baseline and posttraining situation in 8RM test

**SSG**				

	***Pre***	***Post***	**ES**	
**Shoulder Flexion**	157.38 ± 7.67	169.13 ± 7.84	1.05	Moderate
**Shoulder Extension**	34.88 ± 42.0	42.25 ± 4.52^*‡^	1.76	Large
**horizontal shoulder abduction**	35.63 ± 8.15	43.25 ± 8.84^‡^	0.93	Moderate
**horizontal shoulder adduction**	112.38 ± 6.52	119.63 ± 6.16	1.11	Moderate
**torso flexion**	71.88 ± 6.08	86.25 ± 9.32^*^	2.36	Large
**torso extension**	19.63 ± 5.04	26.00 ± 5.12	1.26	Moderate
**hip flexion**	73.25 ± 3.77	96.00 ± 7.52^*^	6.03	Large
**hip extension**	14.50 ± 3.89	20.38 ± 5.07	l.48	Moderate

**PIG**				

**Shoulder Flexion**	160.252 ± 9.59	166.25 ± 10.18	0.62	Small
**Shoulder Extension**	29.25 ± 7.65	31.75 ± 5.82^‡^	0.32	Trivial
**horizontal shoulder abduction**	26.50 ± 1.70	30.00 ± 2.20^†‡^	2.07	Large
**horizontal shoulder adduction**	114.50 ± 4.10	118.00 ± 3.62	0.85	Moderate
**torso flexion**	71.75 ± 9.62	79.25 ± 12.39	0.78	Trivial
**torso extension**	16.13 ± 2.99	20.38 ± 3.16	1.41	Moderate
**hip flexion**	76.38 ± 7.05	93.25 ± 7.99^†^	2.39	Large
**hip extension**	13.88 ± 1.95	17.38 ± 3.02^†^	1.78	Large

* Significant difference between pretraining situation and posttraining in SSG.

† Significant difference between pretraining situation and posttraining in PIG.

**Table 3:** Flexibility results at baseline and posttraining situation

**SSG**				

	***Pre***	***Post***	***ES***	
**Bench Press**	77.88 ± 7.43	88.25 ± 7.48	1.4	Moderate
**Leg Extension**	81.88 ± 9.98	104.63 ± 13.14^*^	2.28	Large
**Low Row**	86.25 ± 10.26	106.25 ± 11.58^*^	1.95	Large
**Leg Curl**	55.38 ± 11.00	66.25 ± 9.91	0.99	Small
**Shoulder Press**	70.25 ± 9.14	79.50 ± 8.40	1.01	Small
**Leg Press**	225.63 ± 44.84	280.63 ± 48.43	1.22	Moderate

**PIG**				

**Bench Press**	76.38 ± 6.34	84.13 ± 6.37	1.22	Moderate
**Leg Extension**	76.00 ± 9.44	94.50 ± 14.48^†^	1.95	Large
**Low Row**	90.13 ± 5.87	107.00 ± 7.50^†^	2.88	Large
**Leg Curl**	51.63 ± 11.40	58.63 ± 13.39	0.61	Small
**Shoulder Press**	66.63 ± 11.55	73.13 ± 12.49	0.56	Small
**Leg Press**	233.13 ± 44.95	279.38 ± 45.62	1.03	Moderate

* Significant difference between pretraining situation and posttraining in SSG.

† Significant difference between pretraining situation and posttraining in PIG.

‡ Significant difference between groups in posttraining situation.

**Table 4:** Hormones responses results at baseline and posttraining situation

	**SSG**			**PIG**		

	***Pre.***	***Post***	***ES***	***Pre***	***Post***	***ES***

**Cortisol**	16.71 ± 5.15	15.70 ± 5.55	0.20 - Trivial	13.64 ±5.61	14.39 ± 5.3	0.13 - Trivial
**Growth Hormone**	0.18 ± 0.14	0.27 ± 0.20	0.09 - Trivial	0.15 ± 0.13	0.19 ± 0.09	0.04 - Trivial

No Significant difference between pretraining situation and posttraining in SSG.

No Significant difference between pretraining situation and posttraining in PIG.
